# Genome-Wide Analysis and Expression Profiling of Lectin Receptor-like Kinase Genes in Watermelon (*Citrullus lanatus*)

**DOI:** 10.3390/ijms25158257

**Published:** 2024-07-29

**Authors:** Duo Lv, Gang Wang, Jiaqi You, Lihua Zhu, Hongjuan Yang, Biting Cao, Weihong Gu, Chaohan Li

**Affiliations:** 1 Shanghai Key Lab of Protected Horticultural Technology, Horticultural Research Institute, Shanghai Academy of Agricultural Sciences, Shanghai 201106, China; 18841608243@163.com (D.L.); youjiaqi@saas.sh.cn (J.Y.); zhulihua.007@163.com (L.Z.); 13501721822@163.com (H.Y.); caobis@163.com (B.C.); 2 School of Agriculture and Biology, Shanghai Jiao Tong University, Shanghai 200240, China; wg770801@sjtu.edu.cn

**Keywords:** Cucurbitaceae, LecRLK, watermelon, RNA-seq, fungal response

## Abstract

Watermelon is one of the most important edible plants worldwide. Owing to its special cultivation conditions, watermelon is exposed to many biological and abiotic stresses during its development. Lectin receptor-like kinases (LecRLKs) are plant-specific membrane proteins that play important roles in sensing and responding to environmental stimuli. Although the *LecRLK* gene family has been identified in a variety of plants, a comprehensive analysis has not yet been undertaken in watermelon. In this study, 61 putative *LecRLK* genes were identified in watermelon, consisting of 36 G-type, 24 L-type, and 1 C-type *LecRLK* genes. They were distributed in clusters on chromosomes, and members from the same subfamily were mostly clustered together. The analysis of the phylogenetic tree and conserved motif indicated that there were obvious differences among three ClaLecRLK subfamilies, and there was also rich diversity in the C-terminal within subfamilies. A collinear analysis revealed that the evolution of the *ClaLecRLK* gene family in different Cucurbitaceae crops was asynchronous. Furthermore, the analysis of the ClaLecRLK protein structure showed that not all proteins contained signal peptides and a single transmembrane domain. A subcellular localization assay confirmed that the number and position of transmembrane domains did not affect ClaLecRLK protein localization in cells. Transcriptome data revealed distinct expression patterns of *LecRLK* genes of watermelon in various tissues, and their responses to different fungi infection were also significantly different. Finally, the potential binding sites of the *ClaLecRLK* genes targeted by miRNA were predicted. This study enhances the understanding of the characteristics and functions of the *LecRLK* gene family in watermelon and opens up the possibility of exploring the roles that *LecRLK* genes may play in the life cycle of Cucurbitaceae plants.

## 1. Introduction

Receptor-like kinases (RLKs) are a class plasma membrane protein that play an important role in mediating the higher plants’ response towards environmental signals [1]. RLKs perceive signals through the extracellular ligand recognition domain and transduce the message to downstream effector molecules via the cytoplasmic kinase domain [2]. On the basis of extracellular domains’ diversity, RLK can be classified into 15 classes [3]. Lectin receptor-like kinase (LecRLK) is a member of RLK, their extracellular domain resembles carbohydrate-binding lectin proteins (also called lectin domains), and, at the same time, LecRLKs also contain at least one transmembrane domain and intracellular kinase domain [2,4]. LecRLKs can be divided into three subfamilies according to their lectin domains: L-, G-, and C-type LecRLKs [2,4]. G-type LecRLKs have the most complex extracellular domain among the three LecRLK types, and often contain the S-locus region, Epidermal Growth Factor (EGF) motif, and Plasminogen Apple Nematode (PAN) motif; this subfamily has also been referred to as B-type LecRLKs due to the resemblance of their extracellular domains to those of bulb lectin protein [2,5]. L-type LecRLKs are characterized by their extracellular domains, which involve the presence of a legume–lectin protein-like extracellular domain present in these proteins [2]. The lectin domain of C-type LecRLKs is a homolog of the calcium-dependent lectin motifs involved in the self- and non-self-recognition and mediation of innate immune responses and pathogen recognition in mammals [6]. The C-type LecRLK is the smallest LecRLK subfamily, with only one or no member in most plants. The transmembrane region is also critical to maintaining the function of LecRLK; sometimes even a single amino acid change in this region would lead to loss of LecRLK’s function [7]. The cytoplasmic kinase domain is the last major structural region of LecRLKs, most of which are Ser/Thr (Serine and Threonine) kinases and a small part are tyrosine (Tyr) kinases [8,9].

Previous studies have suggested that the functions of LecRLKs are closely related to signal sensing and stress responses in plants. Pi-d2, a constitutively expressed G-type LecRLK, confers resistance to the fungus *Magnaporthe grisea* in rice [7]. OsSIT1, an L-type LecRLK, is involved in salt-induced ethylene signaling and negatively regulates salt tolerance in rice [10]. NbLRK1, another L-type LecRLK, can interact with the *Phytophthora infestans* INF1 elicitin and transduces the hypersensitive response (HR) signal [11]. NaLecRLK1 is a G-type LecRLK, which can suppress the *Manduca sexta*-triggered accumulation of salicylic acid and confer jasmonic acid-mediated defense responses against *M. sexta* herbivory in *Nicotiana attenuata* [12].

The Cucurbitaceae family includes some of the most important fruits and vegetables cultivated as crops globally, which represents staple food sources in many developing countries. With advances in biotechnology and sequencing technology, genome data of several Cucurbitaceae plants have been published to date, including plants of the Benincaseae, Cucurbiteae, Sicyoeae, Momordiceae, and Siraitieae tribes [13]. These studies have greatly facilitated research on the evolutionary relationships among Cucurbitaceae plants. As annual or perennial vines, Cucurbitaceae generally have tendrils [13,14], which can assist in plant climbing and growth. At the same time, owing to their sensitive growth rhythm [15] and high vulnerability to pathogen infection [16], Cucurbitaceae plants also have very strict environmental requirements for growth. In particular, signal transduction plays an important role in the growth and development of Cucurbitaceae.

Genome-wide analyses of the *LecRLK* gene family have been reported for some plants [17]; however, a comprehensive understanding of the *LecRLK* genes in watermelon (*Citrullus lanatus*) is lacking. In this study, we used bioinformatics methods to identify *LecRLK* genes from the watermelon genome and analyzed their phylogenetic relationships, gene structure, conserved motifs, gene duplications, chromosome distribution, and collinear relationship with other Cucurbitaceae plants. Finally, we investigated the subcellular localization of LecRLK proteins with different structures, and explored the expression patterns of *LecRLK* family members after inoculation with various fungi using RNA-sequencing (RNA-seq) in watermelon. These results can help to lay the foundation for further in-depth research on the functions of the *LecRLK* gene family in watermelon.

## 2. Results

### 2.1. Genome-Wide Identification of LecRLKs in Watermelon

A total of 61 *LecRLK* genes were identified in watermelon through Pfam (2021), SMART (2020), and CDD (2023) searches, and were named *ClaLecRLK* genes (Table 1). The number of *LecRLK* genes in watermelon was more than that in cucumber (46 *LecRLK* genes), another member of the Benincaseae tribe, but was less than that in *Arabidopsis* (75 *LecRLK* genes) or rice (173 *LecRLK* genes). Based on the diversity of the lectin domain, the 61 *ClaLecRLK* genes can be divided into 36 G-type and 24 L-type *LecRLK* genes, and only 1 C-type *LecRLK* gene.

We further explored the basic characteristics of each *ClaLecRLK* gene and their encoded proteins (Table 1). As for all *ClaLecRLK* genes, the range of coding sequence (CDS) length was 588 to 4557 bp, the range of gene length was 707 to 15,727 bp, the molecular weights (MWs) of the proteins ranged from 63.4 to 171.1 kDa, and the isoelectric points (Ips) ranged from 5.14 to 10.04. Although the average CDS length of L-type *ClaLecRLK* genes (approximately 1968 bp) was longer than that of the C-type *ClaLecRLK* gene (1689 bp), resulting in longer and heavier proteins, the average gene length of L-type *ClaLecRLK* genes (approximately 2221 bp) was shorter than that of the C-type *ClaLecRLK* gene (2533 bp), suggesting that the gene structure of the C-type *ClaLecRLK* gene was more complex than L-type *ClaLecRLK* genes. The genes and CDS length of G-type *ClaLecRLK* genes (approximately 2517 bp and 3682 bp, respectively) were significantly longer than those of C-type and L-type *ClaLecRLK* genes, which can be attributed to the fact that G-type *ClaLecRLK* genes usually contain both EGF and PAN domains. The length of ClaLecRLK protein was 588 to 4557 amino acids, and the molecular weights varied significantly, ranging from 63.4 to 171.1 KDa. The average isoelectric point (pI) of the C-type ClaLecRLK protein was approximately 9.26, whereas the pI of L-type ClaLecRLK protein was approximately 6.33 and that of G-type ClaLecRLK protein was approximately 6.48, showing weak acidity. Although the pI difference between L-type and G-type ClaLecRLK proteins was not significant (*p* = 0.05), the number of G-type ClaLecRLK proteins (seven genes) with a pI greater than 7.0 was greater than the number of L-type ClaLecRLK proteins (two genes).

### 2.2. Chromosomal Location and Gene Duplication of ClaLecRLK Genes

**Figure 1:**
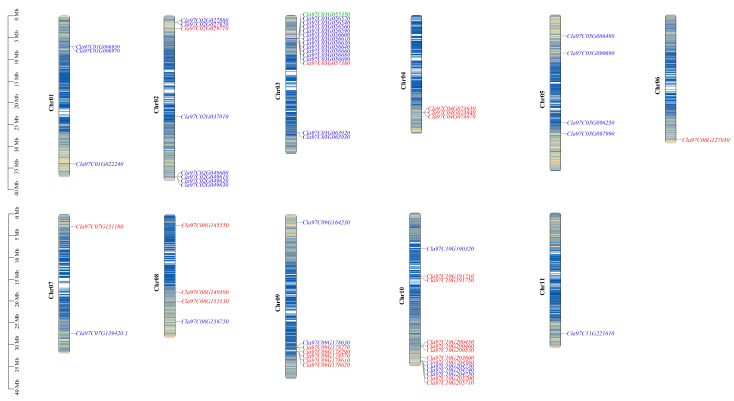
The chromosomal distribution of *ClaLecRLK* genes. The red ID represents L-type *ClaLecRLK* genes, the green ID represents C-type *ClaLecRLK* genes, and the blue ID represents G-type *ClaLecRLK* genes. The genetic distances of seven chromosomes are represented by the scale in megabases (Mb) on the left.

The position of *ClaLecRLK* genes in the genome showed that the members of this family were distributed in clusters and were unevenly distributed on the watermelon’s chromosomes (Figure 1). Most of the *ClaLecRLK* genes were located on chromosome 3 with 15 *ClaLecRLK* genes and chromosome 10 with 13 *ClaLecRLK* genes. On the other hand, the G-type *ClaLecRLK* genes were mainly distributed on chromosome 2 with 7 genes and chromosome 3 with 13 genes. The L-type *ClaLecRLK* genes were mainly distributed on chromosome 9 with six genes and chromosome 10 with nine genes. The C-type *ClaLecRLK* genes were distributed on chromosome 3.

During the long process of evolution, gene duplication (tandem duplication and segmental duplication) is considered to be the main contributor to the generation of gene clusters and expansion of gene families [18,19]. Therefore, we investigated whether gene duplication occurred in the *ClaLecRLK* gene family. To our surprise, only one pair of duplicate genes were identified, *Cla97C03G056520* and *Cla97C03G056540*, and their divergence occurred about 11.85 million years ago. The Ka/Ks value of *Cla97C03G056520* and *Cla97C03G056540* was 0.29, suggesting purifying selection acting on the *ClaLecRLK* gene family. This result indicated that the duplication event of the *LecRLK* gene family during the evolution of the watermelon genome was rare.

### 2.3. Analysis of Phylogenetic and Synteny in ClaLecRLK Genes

**Figure 2:**
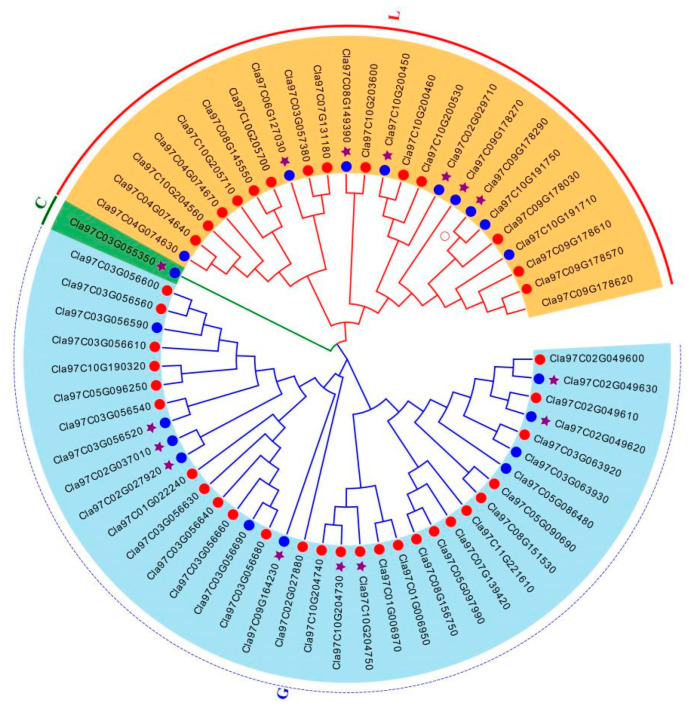
The phylogenetic tree analysis of ClaLecRLK proteins. The L, G, and C, respectively, represent the L-type, G-type, and C-type LecRLK subfamilies. The red circles in front of ClaLecRLK proteins represent proteins with a signal peptide; the blue circles in front of ClaLecRLK proteins represent protein without a signal peptide. The purple star in front of ClaLecRLK proteins represents protein that has at least two transmembrane domains.

Based on the protein sequence of *ClaLecRLK* genes, a phylogenetic tree was constructed to analyze the evolutionary relationship of the ClaLecRLK family (Figure 2). As the same as the cucumber *LecRLK* gene family, although the *ClaLecRLK* gene family can be divided into multiple subgroups, three types were clearly distinguished on the phylogenetic tree; L-type ClaLecRLK and C-type ClaLecRLK groups had a closer evolutionary relationship. Combined with the analysis of protein structure, it was found that ClaLecRLK proteins that distributed in clusters on chromosomes were more likely to cluster into one subgroup than those that were structurally similar. The phylogenetic tree based on the kinase domain showed that three types of ClaLecRLK proteins were still distributed on three different branches (Supplementary Figure S1), but L-type ClaLecRLK and G-type ClaLecRLK were more closely related. This suggested that in addition to the N-terminal lectin domain, the C-terminal kinase domains from different subfamilies were still conserved, and different types of ClaLecRLK protein may have different interacting proteins in cells.

**Figure 3:**
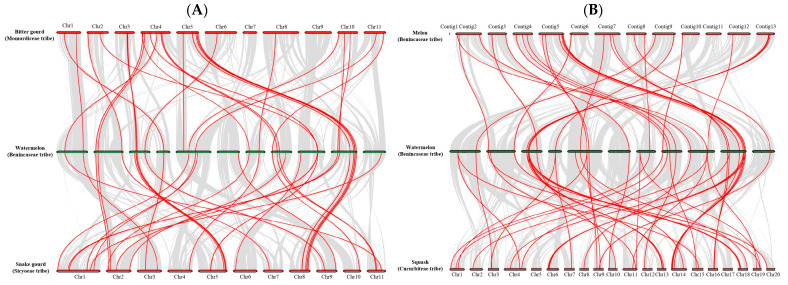
The collinear relationship analysis of the *LecRLK* gene in Cucurbitaceae plants. The collinear gene pairs with *LecRLK* genes between different species are highlighted by the red lines. (**A**) The collinear gene pairs with LecRLK genes among bitter gourd, watermelon, and snake gourd. (**B**) The collinear gene pairs with LecRLK genes among melon, watermelon, and squash.

To further reveal the origin and evolution of *LecRLK* family members in Cucurbitaceae plants, the MCScanX method [20] was used to analyze the collinearity of *LecRLK* genes among the Siraitieae, Momordiceae, Sicyoeae, Benincaseae, and Cucurbiteae tribes (Figure 3 and Supplementary Table S2). We found that 40 *LecRLK* genes had a collinear relationship with the homologous genes of melon and watermelon. There was a collinearity between 42 *LecRLK* genes in watermelon and 53 *LecRLK* genes in squash. At the same time, we also found 43 *LecRLK* genes in watermelon and 44 *LecRLK* genes in snake gourd, having a collinear relationship. On the other hand, there was a collinear relationship between the 33 *LecRLK* gene pairs in watermelon and bitter gourd. Meanwhile, 24 *LecRLK* genes had a collinear relationship with the homologous genes of monk fruit and watermelon. Among them, 10 *LecRLK* genes had a collinear relationship in all six plants (named Cucurbitaceae *LecRLK* genes); these results indicated that these 10 *LecRLK* genes were relatively conserved in the evolution of Cucurbitaceae plants, and the homology of this gene family in Cucurbitaceae has no tribe preference. Finally, we found that only two Cucurbitaceae *LecRLK* genes had a collinear relationship with the homologous genes of rice (Table 2), which indicated that these two *LecRLK* genes were relatively conserved in the evolution of monocots and dicots.

### 2.4. Gene Structure and Protein Motif Analysis of ClaLecRLK Genes

**Figure 4:**
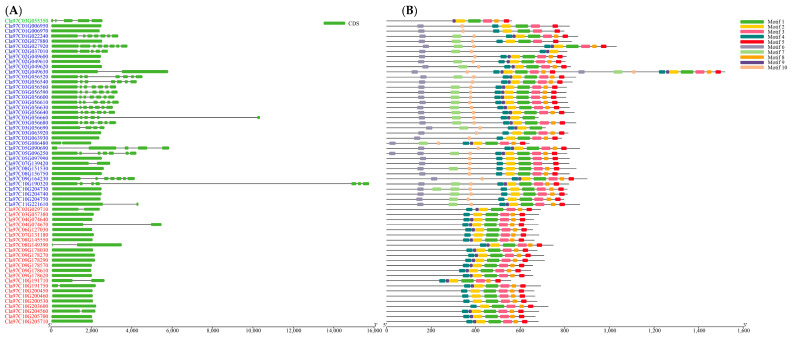
The gene structure and conserved motif analysis of the ClaLecRLK family. (**A**) Gene structure: The lines represent the introns. The green square represents the exon. (**B**) Protein structure: Different colors represent different motifs. The red ID represents L-type *ClaLecRLK* genes, the green ID represents C-type *ClaLecRLK* genes, and the blue ID represents G-type *ClaLecRLK* genes.

The genomic sequence and corresponding cDNA sequence of the *ClaLecRLK* genes were submitted together to the TBtools-II software (v2.001) for analyzing their gene structure (Figure 4). The number of exons of *ClaLecRLK* genes varied from one to eight. Similar to the structure of the *LecRLK* family in other plant genomes, *ClaLecRLK* genes generally lack introns. In total, 85% of the studied *ClaLecRLK* genes had less than three exons. Except for *Cla97C04G074630*, which contained three exons, all L-type *ClaLecRLK* genes contained only one or two exons, and the C-type *ClaLecRLK* gene (*Cla97C03G055350*) contained five exons. The G-type *ClaLecRLK* genes contained one to eight exons. In this group, *Cla97C02G027920* contains eight exons, which has the highest number of exons in all analyzed *ClaLecRLK* genes.

The ten most conserved motifs were identified in ClaLecRLK proteins using the MEME program (Figure 4). The results showed that motif 6, 7, and 10 were only present in G-type ClaLecRLKs, located on the B-Lectin, Pan, and EGF domains, respectively. The remaining seven motifs were located in the kinase domain, and although each motif appeared nearly 50 times, they had 19 different combination forms, of which were G-type ClaLecRLKs containing 16 motif combinations and L-type ClaLecRLKs containing 11 motif combinations. These different motif combinations can increase the diversity of the kinase domain. Motif 2–motif 1–motif 3 was the most conserved motif combination, which appeared in the kinase domain of 41 ClaLecRLKs. In the kinase domain, C-type ClaLecRLKs contained 4 motifs, G-type ClaLecRLKs contained 5.8 motifs, and L-type ClaLecRLKs contained 5.8 motifs, indicating that the conservativeness in the kinase domain of the three types of ClaLecRLKs was similar. By aligning the LecRLK protein sequences of cucumber, rice, and watermelon, it was found that most motifs have been mutated or lost during evolution, and only motif 1, motif 2, motif 3, and motif 5 were relatively intact in these plants, indicating that these four motifs were highly conserved during the evolution of monocots and dicots.

**Figure 5:**
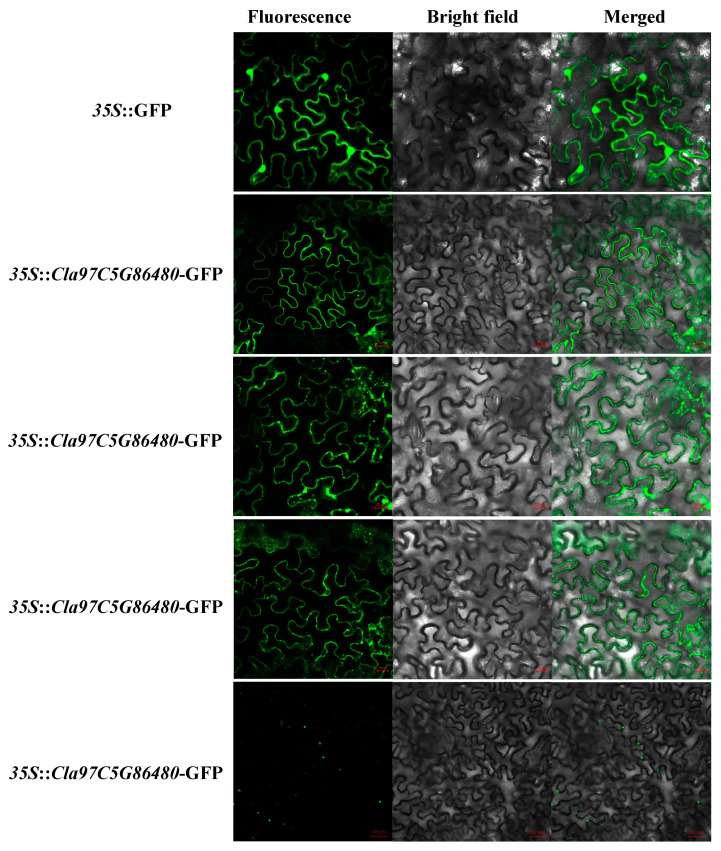
Subcellular localization of ClaLecRLK–GFP fusion proteins in tobacco leaves. Scale bars are 50 µm.

Although bioinformatic predictions suggested that almost all ClaLecRLK proteins were localized on the plasma membrane, the analysis of the protein structure of the ClaLecRLK protein showed that in addition to the three basic domains (N-terminal ligand recognition domain, C-terminal kinase domain, and transmembrane domain), there were various domain differences among LecRLKs, which may affect protein localization. For example, Cla97C09G178270 did not contain a signal peptide, but contained two transmembrane domains located in the N-terminal and middle regions of the protein. Cla97C10G204750 contained a signal peptide and two consecutive transmembrane domains located in the middle of the protein, whereas Cla97C01G022240 contained a signal peptide and transmembrane domain located in the middle of the protein. Subcellular localization experiments showed that these proteins were localized on the cell membrane; Cla97C05G086480, Cla97C01G022240, and Cla97C09G178270 were uniformly expressed on the membrane, whereas Cla97C10G204750 was expressed in a punctate manner (Figure 5).

### 2.5. Response of LecRLK Genes to Fungal Infection in Watermelon

The *LecRLK* gene has been proven in several studies to be involved in responses to biotic stress. During the production process, cucurbitaceae crops are exposed to a variety of fungi, such as *Fusarium oxysporum* f. sp. *niveum*, which is an obligate parasite of watermelon [21]; *Stagonosporopsis* sp, which has a broad spectrum of infection to cucurbit crops [22]; and *Trichoderma koningiopsis*, which can promote plant growth [23]. In this study, transcriptome data were used to analyze the response characteristics of the watermelon *ClaLecRLK* gene family to different fungi.

Under T-51 infection stress, the expression levels of most *ClaLecRLK* genes did not differ significantly in watermelon (Table 3 and Supplementary Table S3). The expression levels of *Cla97C10G203600* and *Cla97C08G151530* were increased by 1.8- and 1.6-fold, respectively, whereas the expression levels of *Cla97C03G057380* and *Cla97C02G049610* were decreased by 3.6- and 18.4-fold, respectively, following infection.

The *ClaLecRLK* genes also responded to *Fusarium* wilt (FW) stress, caused by FON-1 (Supplementary Table S3). The expression levels of 18 *ClaLecRLK* genes were significantly different after inoculation with FON-1, among which only 1 gene was downregulated, whereas the other 17 genes were upregulated, with a change in the expression level ranging from 1.6- to 303.2-fold (Table 3 and Supplementary Table S3). Notably, the expression of *Cla97C03G056680* was barely detected in the roots of the uninfected control group.

**Figure 6:**
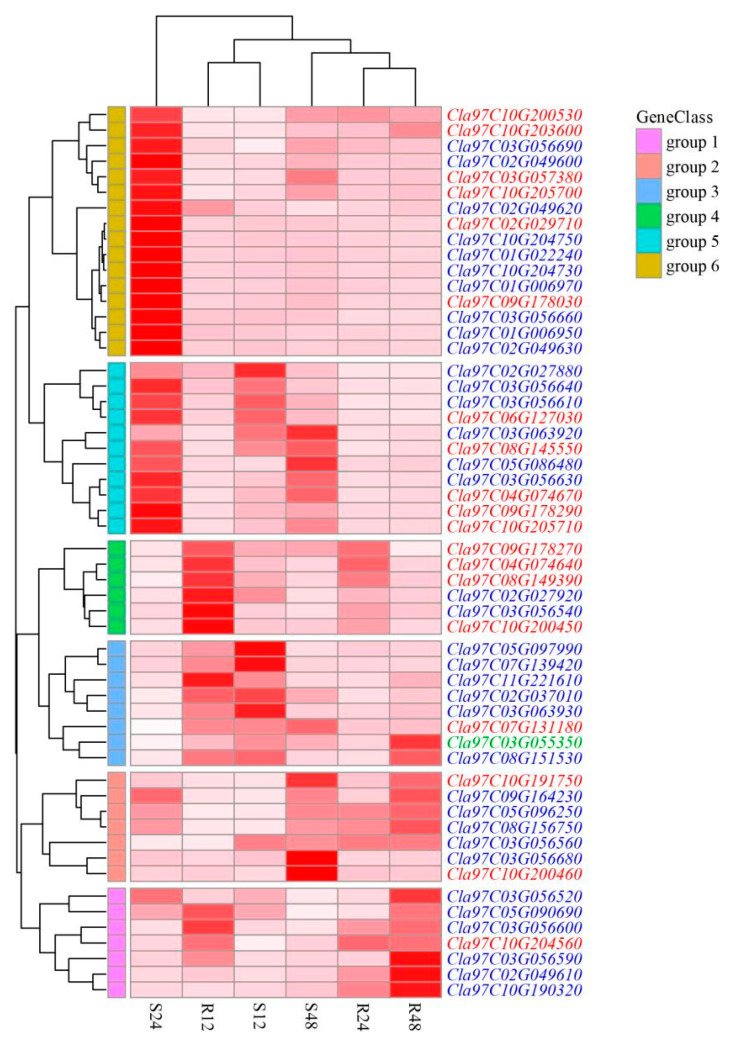
The heatmap of the *ClaLecRLK* gene family to D1320. The expression of *ClaLecRLK* genes is shown on the heatmap using a log2foldchange value. The red ID represents L-type *ClaLecRLK* genes, and the blue ID represents G-type *ClaLecRLK* genes. Genes highly expressed in tissues are colored red, and genes not expressed in tissues are colored white. The letters at the bottom indicate R: resistant material and S: susceptible material. The numbers (12, 24, 48) indicate inoculation time.

**Figure 7:**
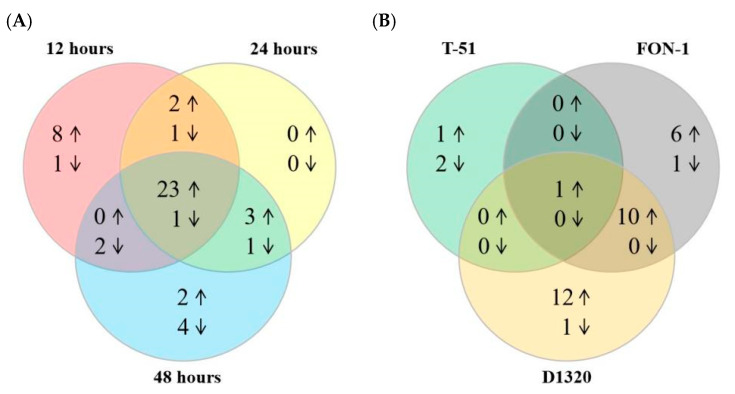
The Venn diagram of differentially expressed *ClaLecRLK* gene response to different fungi. (**A**) Differentially expressed *ClaLecRLK* gene response to D1320 in three time points. (**B**) Differentially expressed *ClaLecRLK* gene response to different fungi. The number in the circle represents the number of differentially expressed genes, the upward arrow indicates genes that are upregulated, and the downward arrow indicates genes that are downregulated.

The transcriptional levels of *ClaLecRLK* genes were investigated at different time points after the infection of D1320. The expression levels of most *ClaLecRLK* genes changed significantly, while some genes showed the same expression trend at different time points (Figure 6 and Supplementary Table S3). After a 12 h inoculation with D1320, there were 33 *ClaLecRLK* genes with upregulated expression and 7 *ClaLecRLK* genes with downregulated expression in susceptible material (W4K-1 inbred lines), and 37 *ClaLecRLK* genes with upregulated expression and 7 *ClaLecRLK* genes with downregulated expression in resistant materials (au-s inbred lines). Interestingly, all upregulated *ClaLecRLK* genes in the susceptible materials were equally significantly upregulated in the resistant material. After a 24 h inoculation, 34 and 35 *ClaLecRLK* genes in resistant and susceptible materials were significantly upregulated, respectively; among them, 28 *ClaLecRLK* genes were upregulated simultaneously. In addition, seven and eight *ClaLecRLK* genes in resistant and susceptible materials were significantly downregulated, respectively. Meanwhile, after a 48 h inoculation, there were 34 *ClaLecRLK* genes that were upregulated in both susceptible and resistant materials, and among them 28 *ClaLecRLK* genes were upregulated in both materials. The Venn diagram shows that a total of 23 *ClaLecRLK* genes were upregulated at all three time points in both resistant and susceptible materials (Figure 7A), indicating that these genes were involved in watermelon response to D1320. Notably, although *Cla97C10G204740* had a low background expression level, it was specifically upregulated in resistant materials at three time points, and *Cla97C10G190320* was specifically upregulated in resistant materials at 24 h and 48 h, suggesting that these two genes might be involved in watermelon resistance to D1320.

The expression levels of 11 *ClaLecRLK* genes were increased after inoculation with FON-1 and D1320 (three time points in both materials) (Table 3 and Figure 7), among which *Cla97C08G151530* expression was also upregulated after inoculation with T-51 (Table 3 and Figure 7B), suggesting that this gene might play an important role in watermelon’s immune response to fungi.

### 2.6. Tissue-Specific Expression of ClaLecRLK Genes

**Figure 8:**
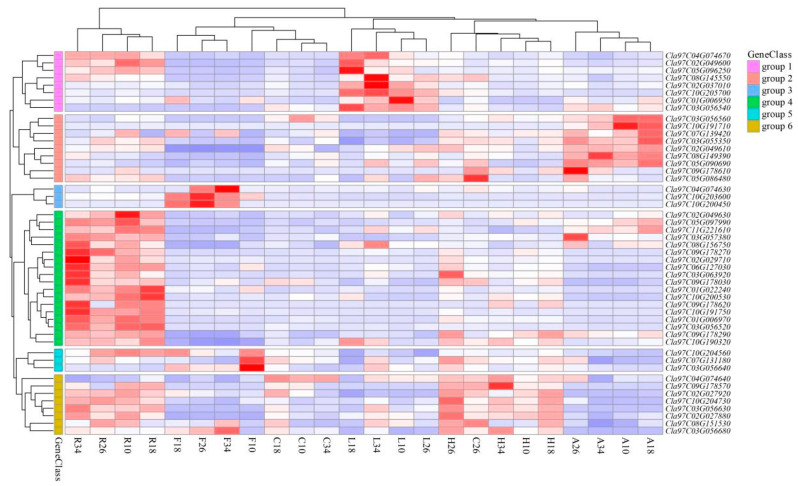
The heatmap of the *ClaLecRLK* gene family in different tissues. The red represents L-type *ClaLecRLK*s, the green represents C-type *ClaLecRLK*s, and the blue represents G-type *ClaLecRLK*s. Genes highly expressed in tissues are colored red, and genes not expressed in tissues are colored blue. The letters at the bottom indicate F: fruit; L: leaf; R: root; A: apical point; C: carpopodium; and H: hypocotyl. The numbers at the bottom indicate the day after pollination.

RNA-seq data (PRJNA532463) from the CuGenDB database were used to investigate the tissue-specific expression of the *ClaLecRLK* gene family. Each tissue contained at least 20 constitutively expressed genes (average FPKM ≥ 1) (Figure 8 and Supplementary Table S4) and 10 *ClaLecRLK* genes were expressed in all tissues at different time points, indicating that the *ClaLecRLK* gene family plays an important role in watermelon development. Some *ClaLecRLK* genes exhibited distinct tissue and temporal expression patterns. For example, *Cla97C01G006970* was highly expressed in the roots (average FPKM ≈ 21.35) but was barely expressed in the fruits and hypocotyls. *Cla97C03G056540* was hardly detected in the fruit, but maintained a relatively high expression level in the leaves (FPKM ≈ 17.46). *Cla97C10G200450* was mainly expressed in the fruit and was significantly upregulated 18 days after pollination. The roots had the most widely expressed *LecRLK* genes, and the average FPKM values of these 38 genes were greater than 1. The fruits had the lowest expression levels of *LecRLK* genes with only 20 genes showing constitutive expression, indicating that the expression of all members of the gene family had a tissue bias.

**Figure 9:**
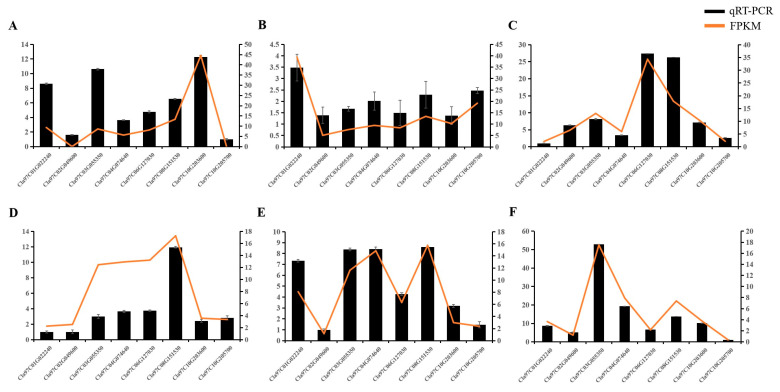
The expression analysis of selected *ClaLecRLK* genes. The orange line represents the value of mean FPKM (fragments per kilobase per million) and the black column represents the expression levels analyzed by qRT-PCR. (**A**): Fruit, (**B**): Leaf, (**C**): Root, (**D**): Apical point, (**E**): Carpopodium, (**F**): Hypocotyl.

Based on these results, we selected eight typical *ClaLecRLK* genes to verify their tissue-specific expression patterns using reverse transcription–quantitative polymerase chain reaction (Figure 9). The expression of most of these genes was consistent with the RNA-seq results, and the expression level of *Cla97C01G022240* was high in the fruits and leaves but lower in other tissues. The expression level of *Cla97C06G127030* was high in the roots but relatively low in other tissues. *Cla97C08G151530, Cla97C03G055350*, and *Cla97C04G074640* were uniformly expressed in all tissues. The expression level of *Cla97C10G203600* was the highest in the fruits but was lower in other tissues. The expression level of *Cla97C10G205700* was the highest in the leaves but was lower in other tissues.

### 2.7. Prediction Analysis of ClaLecRLK Genes Targeted by miRNA

**Figure 10:**
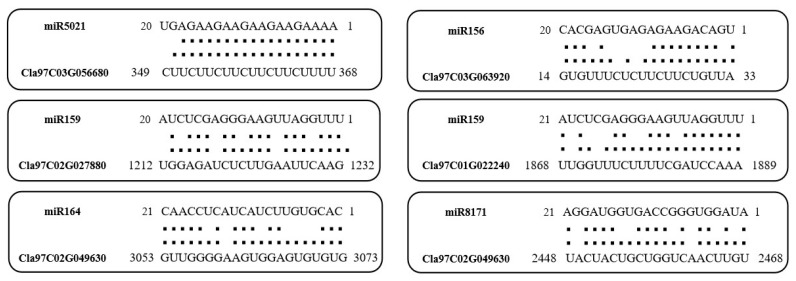
The predicted miRNA information and their binding site on the *ClaLecRLK* genes. One dot indicates that there is a pairing between U and G in the secondary structure, two dots indicate pairing successfully between bases, and a blank space indicates that two bases failed to be paired.

Through the psRNATarget website, we predict the binding sites of published miRNAs targeting the *ClaLecRLK* genes (Supplementary Table S5 and Figure 10). The results showed that a total of 171 miRNAs were involved in the regulation of the *ClaLecRLK* genes. Among 171 miRNAs, except a few miRNAs regulating only one *ClaLecRLK* gene, most of them could regulate multiple *ClaLecRLK* genes. For example, miR5021 had the most regulatory targets, and could regulate 17 *ClaLecRLK* genes; miR156, a famous miRNA in plants, could regulate 8 *ClaLecRLK* genes. In addition, 10 genes included were targeted by miR159. On the other hand, one *ClaLecRLK* gene can also be regulated by multiple miRNAs. For example, *Cla97C02G049630* can be regulated by 25 miRNAs; *Cla97C02G049610* had binding sites of 15 miRNAs.

## 3. Discussion

Generally, LecRLKs are localized on the cell membrane, where they can recognize extracellular signals through their extracellular ligand recognition domains. This helps plants respond accurately and quickly to external stimuli. Utilizing bioinformatics data and biological experiments can provide deeper insights into the signals recognized by LecRLKs and their associated biological pathways. On this basis, integrating new breeding technologies, particularly gene editing, can facilitate faster and more precise breeding for resistance to biotic and abiotic stresses.

At present, genome-wide analyses of the *LecRLK* gene family have been reported for some plants [17,24], but only a few such studies have been reported on Cucurbitaceae crops [4,9]. Therefore, a comprehensive understanding of the *LecRLK* genes of the Cucurbitaceae genome is still lacking. In this study, 61 *LecRLK* genes were identified in watermelon. As for subfamilies, there were 36 G-type *ClaLecRLK* genes, 24 L-type *ClaLecRLK* genes, and just 1 C-type *ClaLecRLK* gene. At the same time, we also counted the number of *LecRLK* genes of different Cucurbitaceae plants, such as melon, *C. maxima*, bitter gourd, and monk fruit; the results showed that they had significant differences in the number of *LecRLK* genes. This suggests that there might not be a direct relationship between *LecRLK* gene family expansion and genome size. According to a previous study [25], chromosome 2 and 3 of watermelon have a collinear relationship with chromosome 1, 2, 3, and 4 of cucumber, and chromosome 9 and 10 of watermelon have a collinear relationship with chromosome 3 and 7 of cucumber. In watermelon, the G-type *ClaLecRLK* genes were mainly distributed on chromosome 2 and chromosome 3, and the L-type *ClaLecRLK* genes were mainly distributed on chromosome 9 and chromosome 10. In cucumber, the G-type *ClaLecRLK* genes were mainly distributed on chromosome 1 and chromosome 4, and the L-type *ClaLecRLK* genes were mainly distributed on chromosome 3 and chromosome 7 [4]. These results suggest that although the *LecRLK* gene family originated from the same ancestors, they experienced relatively independent expansion in different Cucurbitaceae plants.

Most of the conserved motifs (seven motifs) were distributed in C-terminals (kinase domain) of ClaLecRLK proteins, and had 19 combinations in different ClaLecRLK proteins. Each subfamily contained approximately the same amount of motifs, which ensures that each subfamily does not have functional redundancy due to the unicity of the N-terminal. The phylogenetic tree showed that the full-length C-type ClaLecRLK protein was more closely related to L-type ClaLecRLK protein, while the phylogenetic tree based on the kinase domain showed that L-type ClaLecRLK proteins were more closely related to G-type ClaLecRLK proteins, which may be due to the N-terminal of G-type ClaLecRLK proteins often carrying multiple other domains (such as EGF, Pan, and/or S domains), and the kinase domains of C-type ClaLecRLK protein are generally considered to have different kinase activities compared with the other two types of ClaLecRLK proteins [2]. But L-type ClaLecRLK proteins and G-type ClaLecRLK proteins did not cross each other in the branches of the kinase domain phylogenetic tree, indicating that the kinase domain of each ClaLecRLK subfamily is also somewhat conservative. 

After Cucurbitaceae crops experienced four additional genome duplication (WGD) events during their evolution, significant differentiation occurred among different genera [13]. This differentiation is also reflected in the collinearity analysis of the LecRLK gene family among Cucurbitaceae plants, which is positively correlated with their phylogenetic relationships. The number of LecRLK genes that had a collinear relationship between watermelon (Cucurbiteae tribe) and melon (Benincaseae tribe), squash (Cucurbiteae tribe), or snake gourd (Sicyoeae tribe) was significantly higher than the number of genes that had a collinear relationship between watermelon and bitter gourd (Momordiceae tribe) or monk fruit (Momordiceae tribe). On the other hand, the collinear relationship between watermelon and squash was not one to one, and there were only 10 collinearity LecRLK genes among different Cucurbitaceae tribes, again indicating that the evolution of this gene family in different Cucurbitaceae crops was independent. The LecRLK family plays important roles in stress response, microbial associations, and plant development. Relatively independent evolution represents a plant-specific adaptation for the perception and propagation of the extracellular signals [26]. Among 10 Cucurbitaceae collinearity LecRLK genes, only 2 genes had a collinear relationship with the homologous genes of rice. This result is consistent with previous reports that the LecRLK gene family was established before the monocot–dicot split [26]. After the monocot–dicot split, the LecRLK gene family experienced uncoordinated expansion in different crops.

As a kind of cell-surface receptor, LecRLK has been confirmed in previous reports to be associated with various biotic and abiotic stresses. Watermelon, a kind of land-cultivated crop, engages in symbiosis with multiple fungi during its growth. In this study, we selected three types of fungi as research targets to explore the role of LecRLK in watermelon resistance to biotic stress. FW, caused by *Fusarium oxysporum* f. sp. *niveum*, affects water transport efficiency by damaging the vascular system [27]. Gummy stem blight (GSB) is caused by *Didymella bryonia*, which can parasitize many kinds of Cucurbitaceae crops, including watermelon, and cause leaf and vine decay [28]. According to a previous study [29], *Trichoderma koningiopsis* isolate T-51 can improve the immunity of plant hosts to pathogens by activating the immune system. Our RNA-seq data further confirmed that the *LecRLK* family is related to fungal disease resistance. For example, the expression levels of 18 *ClaLecRLK* genes significantly changed after inoculation with FON-1, among which 17 *ClaLecRLK* genes were upregulated. Additionally, the expression levels of 24 genes were significantly upregulated after GSB (D1320) infection, of which 23 genes were upregulated. Combined with the above two sets of data, 11 genes showed upregulated expression after inoculation with the two fungi pathogens, indicating that they may play an important role in disease resistance. The inoculation of T-51, as a promising biocontrol agent, also upregulated the expression levels of two *ClaLecRLK* genes, among which *Cla97C08G151530* was also upregulated under the induction of FW and GSB, indicating that this gene plays a role in the non-specific immune response system of watermelon. The expression levels of *Cla97C03G056640*, *Cla97C03G056680*, *Cla97C09G178030*, and *Cla97C09G178290*, coming from two gene clusters in chromosome 3 and chromosome 9, were all increased after inoculation with both FW and GSB pathogens, indicating that these gene clusters are functionally conserved. However, *Cla97C03G056680* and *Cla97C09G178030* were barely expressed in the non-inoculated roots and leaves, suggesting that some genes of clusters may not be expressed in certain tissues due to redundancy, but are significantly upregulated when performing specific biological functions. On the other hand, FW infects watermelon primarily through the roots, and GSB mainly affects the leaves and stems. Combined with transcriptome data in different tissues, it can be found that ClaLecRLK genes, which were highly expressed in roots, were also mostly responsive to FW inoculation, such as *Cla97C09G178030*, *Cla97C09G178270*, Cla97C09G178290, Cla97C10G190320, and Cla97C10G204730, having relatively higher baseline expression levels in watermelon roots compared to other tissues throughout the life cycle, and they were upregulated following FW inoculation. Similarly, some *ClaLecRLK* genes highly expressed in the leaves were also upregulated after GSB inoculation, such as *Cla97C02G037010*, *Cla97C04G074670*, *Cla97C05G096250*, and *Cla97C08G145550*. This suggests that there is an obvious linear relationship between the tissue specificity of ClaLecRLK genes and their resistance to biological stress. *LecRLK* genes are believed to participate in immunity, development, and reproductive processes via their diverse extracellular ligand domain in plants [30]. Although the sizes of the *LecRLK* gene family vary among plant species, G-type *LecRLK* is the biggest subfamily of the *LecRLK* gene in most plants; they are believed to expand over evolutionary time in response to pathogen pressure [31]. In this study, we found that G-type *ClaLecRLK* genes respond more strongly to FW and GSB inoculation compared to the other two *ClaLecRLK* gene subfamilies, indicating that G-type *LecRLK* genes may primarily be involved in sensing pathogen signals. L-type *ClaLecRLK* genes have significantly higher expression in fruits than G-type and C-type *ClaLecRLK* genes, suggesting that L-type *LecRLK* genes are mainly involved in the development of plant organs. C-type *LecRLK* genes have always been a mysterious subfamily because there are usually only one or two of them in most plants, and their mutants are rarely discovered. Recent reports indicate that C-type *LecRLK* genes are involved in the development of trichomes [32] and resistance to aphids [33] in cucumber. These results indicate that each subfamily has a distinct role in the plant’s life cycle.

miRNA (microRNA) is a kind of widely existing, non-coding small RNA with regulatory function in plants and animals. In plants, they are widely involved in regulating growth and development, signal transduction, and stress response [34]. At present, the research of some star miRNAs has been very in-depth, such as miR156, miR160, miR171, and so on. Despite the fact that most of *LecRLK* genes’ function has not yet been studied extensively, analyzing correspondences of miRNAs and genes will provide clues. In our study, most miRNA could regulate multiple *ClaLecRLK* genes. For example, miR156 can affect pollen development and adventitious root formation by promoting cell proliferation [35], and could regulate eight *ClaLecRLK* genes, suggesting that these *ClaLecRLK* genes may be involved in the regulation of plant development. The C-type *LecRLK* gene is the most mysterious subfamily, with only one member in most plants, and studies on its function are very rare. In our study, only one C-type *LecRLK* was identified in watermelon, and it was found that it may be the target of four miRNAs. Among these miRNAs, miR396 targets six Growth-Regulating Factor genes with roles in plant leaf growth in Arabidopsis [36], and miR773 is involved in response to fungal pathogens in Arabidopsis [37]. These results may provide a strong basis for us to further explore the function of the C-type *LecRLK*.

## 4. Materials and Methods

### 4.1. Plant Materials and Fungal Culture

The watermelon inbred line ‘W28-7’ was used to investigate the spatiotemporal expression and response of *ClaLecRLK* genes to *Trichoderma koningiopsis* isolate T-51 and *Fusarium oxysporum* f. sp. *niveum* race 1 (FON-1). The watermelon inbred lines ‘4k-1’ (susceptible material) and ‘au-s’ (resistant material) were used to investigate the response of *ClaLecRLK* genes to *Stagonosporopsis* sp. D1320. Seedlings of watermelon were grown in an incubator without any pathogen (16/8 h light–dark cycle, 25 °C, humidity was 70%) prior to treatment. When the first true leaf fully unfolded, the seedlings were placed in a glass greenhouse for further cultivation, and the controlled environment growth chamber was programed for 16 h light at 25 °C and 8 h dark at 20 °C. The strains of FW (FON-1) and *Stagonosporopsis* sp. D1320 in this study came from the experimental field of our laboratory (Shanghai, China), and have been separated and purified for many generations, which have good infectivity to the experimental materials, and ensure that there is no contamination of stray bacteria or fungi.

The *Trichoderma koningiopsis* isolate T-51 was cultured on solid potato dextrose agar (PDA) plates at 20 °C for 10 days under light. The conidia were washed from the culture using sterilized water and the conidial concentration was determined using a hemocytometer and adjusted to 1 × 10^7^ conidia/mL. *Fusarium oxysporum* f. sp. *niveum* race 1 (FON-1) was cultured on a solid PDA medium, inoculated into a 500 mL liquid PDA medium, and incubated with shaking at 25 °C for 10 days. The resulting mixture was filtered through a four-layer cheesecloth to remove mycelial fragments from the conidial suspensions. The conidial concentration was determined using a hemocytometer and adjusted to 1 × 10^6^ conidia/mL. *Stagonosporopsis* sp. D1320 was cultured on a PDA solid medium at 25 °C for 7 days in the dark. All strains used in this study were obtained from our laboratory’s preservation.

### 4.2. Identification of LecRLK Genes in Different Plants

The whole-genome and protein sequence data of different Cucurbitaceae plants were downloaded from a public Cucurbitaceae database (http://cucurbitgenomics.org/v2/ (accessed on 11 September 2023)), including watermelon (*Citrullus lanatus*, 97103 genome v2.5, accessed on 11 September 2023), bitter gourd (*Momordica charantia*, Dali-11 genome v1, accessed on 11 September 2023), squash (*Cucurbita moschata*, Rifu genome v1, accessed on 16 October 2017), melon (*Cucumis melon*, DHL92 genome v4, accessed on 11 September 2023), monk fruit (*Siraitia grosvenorii*, Qingpiguo genome v1, accessed on 28 December 2021), and snake gourd (*Trichosanthes cucumerina*, genome v1, accessed on 11 May 2021). The *LecRLK* family sequence data of rice and cucumber were obtained from the literature [2,4].

The hidden Markov model (HMM) was used to identify *LecRLK* candidates, and the HMM profiles of the LecRLKs were downloaded from the Pfam protein database (http://pfam.xfam.org/ (accessed on 11 May 2022)) [38]. The models used for these files were L-type (Lectin_legB PF00139), G-type (B_lectin PF01453), and C-type (Lectin_C PF00059). We used HMMER 3.0 [39] to search for the three types of *LecRLK* genes from different plant protein sequence data with an e-value cutoff of <0.001. We then submitted the whole-protein sequences of these genes to two bioinformatics websites, Pfam [40] (http://pfam.xfam.org/ (accessed on 11 May 2022)) and SMART [41] (http://smart.embl.de/ (accessed on 26 October 2020)), with an e-value cutoff of 1.0, while retaining genes that contained the lectin, transmembrane, and kinase domains.

### 4.3. Analysis of Phylogenetic and Gene Structure

The full-length and kinase domain sequences of ClaLecRLK proteins were aligned using the MUSCLE program (http://www.ebi.ac.uk/Tools/msa/muscle/ (accessed on 11 October 2023)) with the default parameters [42]. The Neighbor-Joining (NJ) method contained in MEGAX_software v.10.1.8 was used to construct phylogenetic trees with the following parameters: the Poisson model, pairwise deletion, and 1000 bootstrap replications. TBtools-II (v2.001) was used to show the exon–intron structures of *ClaLecRLK* genes.

### 4.4. Tissue-Specific Expression Pattern Analysis and Subcellular Localization

Total RNA (contained clone of genes) was extracted using the RNeasy Plant Mini Kit (Cwbio, Beijing, China) from plants of watermelon. First-strand cDNA was prepared using a PrimeScript RT Reagent Kit with gDNA Eraser (Cwbio).

Public transcriptome data (PRJNA532463) [25], obtained from Cucurbit Expression Atlas Cucurbit Genomics Database (CuGenDB), were used to investigate expression patterns of the *ClaLecRLK* gene family. The spatiotemporal expression patterns of selected *ClaLecRLK* genes were confirmed by qRT-PCR in multiple tissues, including the roots (4-week-old seedlings), hypocotyls (4-week-old seedlings), apical point (10-week-old seedlings), carpopodium (10-week-old cucumber plants), fruits (unfertilized 10-week-old cucumber plants), and young leaves (10-week-old cucumber plants, from the top node). Three biological replicates were established in this experiment, and every tissue sample was collected from at least eight plants in each replicate.

The full-length cDNA sequences of different *ClaLecRLK* genes were cloned and fused to a pHB vector (*CaMV35S–GFP*). Complete vectors were injected into tobacco (*Nicotiana tabacum*) leaf epidermal cells using the *Agrobacterium*-mediated method [43]. Two days after injection, the green fluorescence signal was detected using an Olympus BX51 fluorescence microscope to acquire fluorescent images. Green fluorescent protein (GFP) imaging was performed at an excitation wavelength of 488 nm. At least three independent replicates were performed and the results of one representative experiment are shown. The primers used are listed in Supplementary Table S1.

### 4.5. Chromosome Localization and Duplication Analyses

A series of in-house Perl scripts were used to retrieve the location information for the *ClaLecRLK* gene and the length of each chromosome from the whole-genome sequence data of watermelon downloaded from the Cucurbitaceae public database (http://cucurbitgenomics.org/v2/ (accessed on 11 October 2023)). According to the gene position, the chromosome locations of the ClaLecRLK genes were mapped using TBtools-II (v2.001). Adobe Illustrator CS6 was used to enhance the images for a detailed analysis.

We used two methods to identify duplication events among the *LecRLK*s in watermelon. In the first method, gene duplication was confirmed according to the following three criteria using in-house Perl scripts: (a) the shorter aligned gene covers >70% of the length of the longer gene, (b) the similarity of aligned sequences is >70% [44], and (c) two genes located in the same chromosomal fragment of less than 100 kb represent tandem duplicated genes [18]. The second method was based on the Multiple Collinearity Scan toolkit (MCScanX) to analyze gene duplication events with default parameters [20].

The synonymous substitution (Ks) and nonsynonymous substitution (Ka) rates of tandemly duplicated genes were calculated using the method of Nei and Gojobori as implemented in the KaKs_calculator [45] based on the coding sequence alignments. The divergence time was calculated based on the formula T = Ks/2r, where Ks is the synonymous substitution rate per site and r is the rate of divergence of nuclear genes from plants; r was taken to be 1.5 × 10^−8^ synonymous substitutions per site per year for dicotyledonous plants [46].

### 4.6. Analysis of Conserved Motif and Synteny

The conserved motif prediction of all ClaLecRLK proteins was performed using the MEME program v.5.1.1 [47] (http://meme-suite.org/ (accessed on 11 October 2023)). The parameters were set as any number of repetitions and an optimum motif width of 10–210 residues. The maximum search time was set as 36,000 s, and the site distribution mode was ‘zoops’. The syntenic analyses were constructed by a series of the R program (v4.2.2) to exhibit the collinear relationship of the orthologous *LecRLK* genes among different plants.

### 4.7. RNA-Seq of Fungal Inoculation

For inoculation with T-51 and FON-1, the watermelon seedlings were root-dipped into the conidial suspension for 30 min and all seedlings were replanted in sterilized soil in a greenhouse. The roots were subjected to RNA-seq 2 and 10 days after T-51 and FON-1 inoculation, respectively. For inoculation with D1320, a mycelial plug (5 mm diameter) from the PDA plate was inoculated on the first true leaf of watermelon seedlings, followed by cultivation at 25 °C and relative humidity > 99%. The first true leaves were subjected to RNA-seq 0, 12, 24, and 48 h after D1320 inoculation. The high-quality RNA samples were sequenced by Biomarker Bioinformatics Technology Co., Ltd. (Beijing, China), and the cDNA library was subjected to high-throughput sequencing (RNA-seq) on the Illumina HiSeq™ 2500 platform. Raw data were deposited in the SRA of the NCBI website under accession numbers PRJNA995348 and PRJNA995420. The methods of the sequence assembly and differential expression gene analysis for RNA-seq used in this study have been described previously [32]. Tissue-specific expression and fungi inoculation analyses of watermelon *LecRLK* genes were performed and the results were visualized as a heatmap using the R program (4.2.2).

### 4.8. Prediction of Binding Sites of the ClaLecRLK Genes Targeted by miRNA

The miRNA sequences were downloaded from psRNATarget (https://www.zhaolab.org/psRNATarget/ (accessed on 16 September 2023)) [48]. The selected cDNA library is ‘Citrullus lanatus (watermelon), transcript, Cucurbit Genomics Database, version 1’, and other parameters are set to default values.

## 5. Conclusions

A total of 61 LecRLK genes of watermelon were identified in our study. Members of the *ClaLecRLK* gene family are distributed in clusters on chromosomes, and members of the same subfamily are mostly clustered together. The results of the phylogenetic tree and conserved motif analysis revealed that in addition to the N-terminal kinase domain, the C-terminal kinase domain was also conserved in different subfamilies. The synteny analysis showed that the *ClaLecRLK* gene family may have been established before the monocot–dicot split, and also has undergone relatively independent evolution in Cucurbitaceae plants. Transcriptome data demonstrated distinct expression patterns of ClaLecRLK genes in various tissues and response to multiple fungal pathogens. The prediction of mirNA-targeted binding sites revealed that each *ClaLecRLK* gene may be regulated by at least three miRNAs. In conclusion, the information obtained in our study could help to provide an in-depth understanding of basic features of the *ClaLecRLK* gene family; this knowledge could be applied to enhance breeding programs and improve management strategies for biotic and abiotic stress resistance in watermelon.

## Figures and Tables

**Table 1:** The basic character of *ClaLecRLK* genes and their encoded proteins.

	Gene ID	Gene	CDS	Protein	MW	pI	Localization	Exon	Intron
		(bp)	(bp)	(aa)	(KDa)				
C-type	*Cla97C03G055350*	2533	1689	562	63.4	9.26	PM	5	4
L-type	*Cla97C02G029710*	2349	2076	691	77.7	6.25	PM	2	1
	*Cla97C03G057380*	2052	2052	683	75.7	6.13	PM	1	0
	*Cla97C04G074630*	707	588	195	22.1	10.04	PM	3	2
	*Cla97C04G074640*	1983	1983	660	73.4	6.66	PM	1	0
	*Cla97C04G074670*	5405	2046	681	76.2	6.49	PM	2	1
	*Cla97C06G127030*	1971	1971	656	73.2	5.16	PM	1	0
	*Cla97C07G131180*	2055	2055	684	76.0	5.58	PM	1	0
	*Cla97C08G145550*	1992	1992	663	73.8	6.12	PM	1	0
	*Cla97C08G149390*	3450	2247	748	83.0	6.74	PM	2	1
	*Cla97C09G178030*	2031	2031	676	74.9	5.44	PM	1	0
	*Cla97C09G178270*	2121	2121	706	79.7	6.18	PM	1	0
	*Cla97C09G178290*	2130	2130	709	79.2	6.22	PM	1	0
	*Cla97C09G178570*	1971	1971	656	73.2	5.93	PM	1	0
	*Cla97C09G178610*	1944	1944	647	73.1	6.99	PM	1	0
	*Cla97C09G178620*	1974	1974	657	73.6	5.89	PM	1	0
	*Cla97C10G191710*	2602	1677	558	62.5	6.27	PM	2	1
	*Cla97C10G191750*	2162	2079	692	77.3	5.97	PM	2	1
	*Cla97C10G200450*	1989	1989	662	74.2	7.92	PM	1	0
	*Cla97C10G200460*	2001	2001	666	74.4	5.88	PM	1	0
	*Cla97C10G200530*	2028	2028	675	75.2	5.32	PM	1	0
	*Cla97C10G203600*	2178	2178	725	78.2	5.79	PM	1	0
	*Cla97C10G204560*	2148	2052	683	74.9	6.45	PM	2	1
	*Cla97C10G205700*	2004	2004	667	74.2	6.65	PM	1	0
	*Cla97C10G205710*	2046	2046	681	75.2	5.78	PM	1	0
	Range	707–5405	588–2247	195–748	22.1–83	5.16–10.04		1–3	1–2
	Average	2221	1968	655	73.0	6.33		1.3	0.3
G-type	*Cla97C01G006950*	2466	2466	821	91.6	8.37	Ec	1	0
	*Cla97C01G006970*	2394	2394	797	88.9	8.72	Ec	1	0
	*Cla97C01G022240*	3318	2580	859	96.2	5.30	PM	7	6
	*Cla97C02G027880*	2493	2493	830	92.3	6.77	PM	1	0
	*Cla97C02G027920*	3740	3096	1031	116.2	5.65	PM	8	7
	*Cla97C02G037010*	2772	2433	810	92.0	8.35	PM	5	4
	*Cla97C02G049600*	2424	2424	807	90.1	5.70	PM	1	0
	*Cla97C02G049610*	2409	2409	802	90.1	5.90	PM	1	0
	*Cla97C02G049620*	2481	2481	826	92.7	6.00	PM	1	0
	*Cla97C02G049630*	5749	4557	1518	171.1	6.41	Ec	2	1
	*Cla97C03G056520*	4511	2553	850	96.3	7.37	PM	7	6
	*Cla97C03G056540*	4213	2505	834	94.0	6.87	PM	7	6
	*Cla97C03G056560*	3185	2427	808	90.2	5.78	PM	7	6
	*Cla97C03G056590*	3255	2421	806	91.5	7.49	PM	7	6
	*Cla97C03G056600*	3111	2418	805	90.7	5.95	PM	7	6
	*Cla97C03G056610*	3314	2445	814	92.1	6.17	PM	7	6
	*Cla97C03G056630*	3007	2466	821	92.8	6.19	PM	7	6
	*Cla97C03G056640*	3119	2532	843	95.7	7.99	PM	7	6
	*Cla97C03G056660*	10,301	2049	682	77.0	5.86	PM	6	5
	*Cla97C03G056680*	3185	2553	850	95.9	6.00	PM	7	6
	*Cla97C03G056690*	2598	2145	714	80.8	7.77	PM	4	3
	*Cla97C03G063920*	2448	2448	815	91.2	5.20	PM	1	0
	*Cla97C03G063930*	2361	2361	786	89.8	5.88	PM	1	0
	*Cla97C05G086480*	1979	1926	641	72.5	6.12	PM	2	1
	*Cla97C05G090690*	5800	2604	867	95.4	5.83	PM	5	4
	*Cla97C05G096250*	4180	2433	810	90.7	6.78	PM	7	6
	*Cla97C05G097990*	2466	2466	821	90.3	5.74	PM	1	0
	*Cla97C07G139420*	2869	2460	819	92.3	7.99	PM	2	1
	*Cla97C08G151530*	2556	2556	851	92.7	5.94	PM	1	0
	*Cla97C08G156750*	2466	2466	821	90.5	5.70	PM	1	0
	*Cla97C09G164230*	4117	2703	900	102.6	6.08	PM	7	6
	*Cla97C10G190320*	15,727	2457	818	92.6	8.21	PM	7	6
	*Cla97C10G204730*	2436	2436	811	91.1	5.74	PM	1	0
	*Cla97C10G204740*	2439	2439	812	91.3	5.14	PM	1	0
	*Cla97C10G204750*	2391	2391	796	89.5	6.19	PM	1	0
	*Cla97C11G221610*	4269	2604	867	95.6	6.08	PM	2	1
	Range	1979–15,727	1926–4557	641–1518	72.5–171.1	5.14–8.72		1–8	0–7
	Average	3682	2517	838	94.1	6.48		3.90	2.90
Range		707–15,727	588–4557	195–1518	63.4–171.1	5.14–10.04			
Average		3088	2287	761	85.3	6.46			

**Table 2:** Collinear relationship analysis of *LecRLK* gene.

Species	Dicot						Monocot
	Bitter Gourd	Snake Gourd	Monk Gourd	Melon	Watermelon	Squash	Rice
Tribe	Momordiceae	Sicyoeae	Siraitieae	Benincaseae	Benincaseae	Cucurbiteae	
Gene ID	MC02G0084	Tan0002591	Sgr014680	MELO3C014624	Cla97C02G049630	CmoCh20G000680	
	MC03Gnew0349	Tan0014355	Sgr029435	MELO3C002524	Cla97C03G056520	CmoCh13G007160	
	MC03G0790	Tan0016767	Sgr019525	MELO3C002606	Cla97C03G057380	CmoCh13G006610	
						CmoCh18G003480	
	MC01G0434	Tan0008913	Sgr026531	MELO3C003146	Cla97C04G074670	CmoCh07G010510	
	MC11G0930	Tan0015648	Sgr028234	MELO3C018168	Cla97C05G096250	CmoCh01G012240	Os01G0784700
						CmoCh09G009010	Os04G0633200
	MC08G0987	Tan0015848	Sgr015781	MELO3C017881	Cla97C07G139420	CmoCh01G001500	
	MC04G1328	Tan0003615	Sgr014359	MELO3C008394	Cla97C08G149390	CmoCh04G025490	
	MC10G0972	Tan0002296	Sgr020073	MELO3C016574	Cla97C10G190320	CmoCh16G008700	Os05G0501400
	MC10G0515	Tan0018666	Sgr020674	MELO3C014953	Cla97C10G191750	CmoCh04G009160	
						CmoCh16G007980	
	MC05Gnew0379	Tan0005166	Sgr022715	MELO3C009237	Cla97C10G204560	CmoCh06G002850	
						CmoCh14G001210	

**Table 3:** Response of differentially expressed *ClaLecRLK* genes to fungi.

Subfamily	Gene ID	Fungi			Regulated Direction	
		T51	FON-1	D1320	Up	Down
L-type	Cla97C10G205710		✔	✔	✔	
	Cla97C09G178290		✔	✔	✔	
	Cla97C02G029710			✔	✔	
	Cla97C04G074670			✔	✔	
	Cla97C08G145550			✔	✔	
	Cla97C10G200450			✔	✔	
	Cla97C09G178030		✔	✔	✔	
	Cla97C10G191750			✔	✔	
	Cla97C09G178270		✔		✔	
	Cla97C10G205700		✔		✔	
	Cla97C10G203600	✔				
	Cla97C08G149390		✔			✔
	Cla97C03G057380	✔				✔
G-type	Cla97C05G096250		✔	✔	✔	
	Cla97C01G022240		✔	✔	✔	
	Cla97C02G037010		✔	✔	✔	
	Cla97C02G027880		✔		✔	
	Cla97C03G056660		✔		✔	
	Cla97C03G056640		✔	✔	✔	
	Cla97C08G151530	✔	✔	✔	✔	
	Cla97C10G204750			✔	✔	
	Cla97C01G006970			✔	✔	
	Cla97C10G204730		✔	✔	✔	
	Cla97C03G063920			✔	✔	
	Cla97C09G164230			✔	✔	
	Cla97C02G049610			✔	✔	
	Cla97C03G063930			✔	✔	
	Cla97C03G056630			✔	✔	
	Cla97C05G086480		✔	✔	✔	
	Cla97C03G056680		✔	✔	✔	
	Cla97C03G056690		✔		✔	
	Cla97C10G190320		✔		✔	
	Cla97C08G156750			✔		✔
	Cla97C02G049610	✔				✔

## Data Availability

The original contributions presented in the study are publicly available. These data can be found at the National Center for Biotechnology Information (NCBI) BioProject database under accession number PRJNA995348 and PRJNA995420.

## Supplementary Materials

The following supporting information can be downloaded at: https://www.mdpi.com/article/10.3390/ijms25158257/s1.
