# Supplementary figures and images for: Genome-Wide Analysis and Expression Profiling of Lectin Receptor-like Kinase Genes in Watermelon (Citrullus lanatus)
Source: Int J Mol Sci. 2024 Jul 29;25(15):8257. doi: 10.3390/ijms25158257 (PMC11312183; doi:10.3390/ijms25158257)

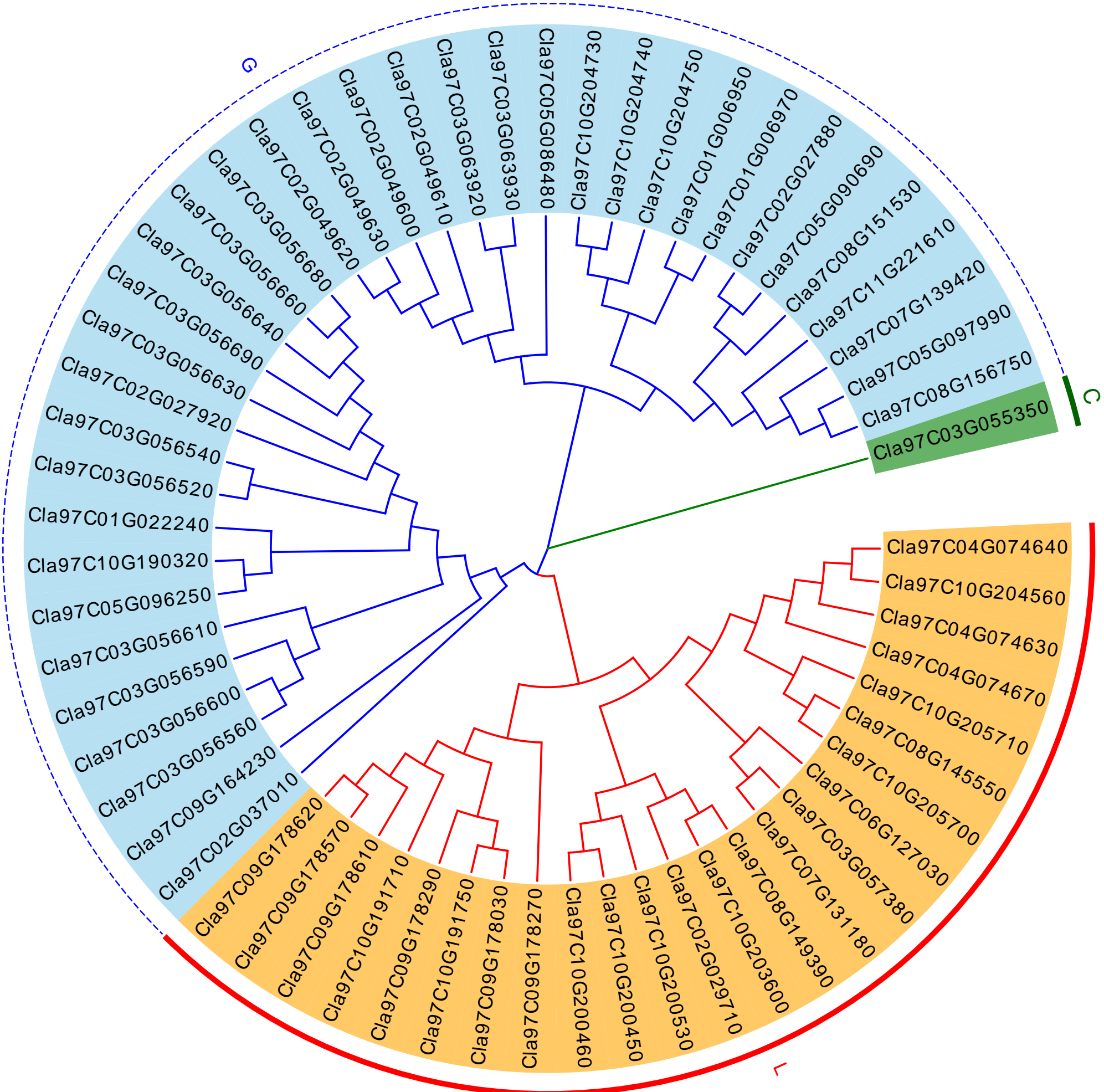

Supplement: Supplementary file 1 [file ijms-25-08257-s001.zip › Supplyment Figure S1.pdf]
